# Feasibility assessment of an EHR-integrated research platform for prospective data collection in community oncology practice

**DOI:** 10.1038/s44401-025-00066-9

**Published:** 2026-01-28

**Authors:** Majd T. Ghanim, Daniel Sanchez, Claire Dykas, Fredrik Borgsten, Abhishek Dabral, Mariana Fernandez, Laura Hester, Maneet Kaur, Lakshmikanth Katragadda, Yichen Lu, Amanda Rodriguez-Sullivan, Melanie Rothschild, Paul M. Salcuni, Natalie Salituro, Richard M. Zuniga, Neal J. Meropol, Ashita S. Batavia

**Affiliations:** 1 Flatiron Health, New York, NY USA; 2 Johnson & Johnson, New Brunswick, NJ USA; 3 Johnson & Johnson, Raritan, NJ USA; 4 Johnson & Johnson, Madrid, Spain; 5 Clearview Cancer Institute, Huntsville, AL USA; 6 New York Cancer and Blood Specialists, Babylon, NY USA

**Keywords:** Cancer, Health care, Medical research, Oncology

## Abstract

Post-approval observational studies that rely on traditional data collection approaches may be burdensome for community oncology settings with limited research infrastructure. We conducted a prospective observational study using an EHR-integrated platform to evaluate its feasibility for streamlining data collection in community oncology. We were able to capture high-quality data on infusion-related reactions in patients with multiple myeloma receiving daratumumab. The EHR-integrated platform included centralized patient identification, automated EHR-to-electronic data capture system data transfer, and centralized abstraction services. Over 11 months, 82 patients enrolled at four US community oncology sites, with 30.5% identifying as Black or Hispanic. Across 4568 essential data points: missingness accounted for 0.59%, 91% of data points were eligible for automated transfer, median transfer time for structured data was 1.7 days, and query frequency was <2% with a median resolution time of 5.1 days. This pilot study demonstrated that EHR-integrated platforms can be operationalized in community oncology settings with high data completeness and rapid, diverse enrollment, expanding access to clinical trials outside traditional research centers and producing more generalizable results.

## Introduction

Post-approval studies of cancer therapies are often conducted to address ongoing gaps in evidence. These studies may be overseen by the US Food and Drug Administration (FDA) as Postmarketing Commitments (PMCs) or Postmarketing Requirements (PMRs) to collect additional data regarding safety and efficacy of a treatment^1^. In addition to regulatory considerations, post-approval studies may be used to inform clinical decisions in routine practice^2^, establish registries with linked clinical and biologic data for discovery or clinical validation, or support payer access decisions^3^. Post-approval studies may also characterize long-term outcomes^4^, alternate dosing or treatment schedules^5^, or expanded indications^6^. Resource-intensive data collection requirements are associated with delays in completion of post-approval studies for patients with cancer, even those that are required by the FDA^7^. Current challenges to the timely and successful completion of post-approval studies for patients with cancer include complex data collection, limited site capacity for conducting research studies, constraints on resources, and lower prioritization by site investigators that favor interventional studies of novel agents^8^. New design and operational approaches to post-approval studies that streamline participation for patients and study sites can enable evidence generation and ensure access for participants wherever they receive care.

Historically, many post-approval studies are designed and conducted with traditional clinical trial operational models that include heavy data collection burden, and take place at specialized research centers (e.g., academic medical centers) that limit the representativeness of the patients who are enrolled^9^. Traditional methods of data capture require manual transfer from the electronic health record (EHR) and other data sources into a separate electronic data capture (EDC) system. This process increases the potential for transcription errors, introduces inconsistencies between source and the study records, and requires substantial site resources. Adding to site burden, the data required for PMCs and PMRs often exceeds what is routinely documented in clinical care. These barriers are particularly acute in community settings, where research infrastructure is often limited^10^. In addition, patient identification processes are labor-intensive and typically rely on investigator-clinician referrals or manual chart review, which can be inefficient, inconsistently applied, and prone to selection bias^11^. Traditional processes tend to favor academic medical centers that have more robust research infrastructure including support staff and dedicated non-clinical research time for investigators. This has historically contributed to geographic and demographic disparities in patient access and participation and enrollment of less representative patient populations^12,13^.

Given that EHRs are widely used for documenting cancer care and much of the data needed for clinical studies already exists within these systems^14^, we developed a clinical study platform to seamlessly integrate research processes into EHR workflows. This platform supports more efficient conduct of post-approval studies in community settings. Key features of the platform include automated patient-matching tools to assist with real-time identification of eligible patients, and a Fast Healthcare Interoperability Resources (FHIR)-based EHR-to-EDC connector for automated transfer of EHR clinical data, including direct entry of study-specific data elements (e.g., adverse events [AEs]) within routine clinical workflows. Health Level Seven International (HL7) FHIR and proprietary application programming interfaces (APIs) are increasingly being recognized as enabling technologies for EHR-to-EDC data transfer^15–19^; however, to date significant adoption challenges exist^18^. In this report, we described a pilot implementation study of a technology-enabled platform for post-approval evidence generation in oncology. The clinical context for this study is collection of infusion-related AEs in patients with multiple myeloma who are treated with daratumumab. Daratumumab is a monoclonal antibody that targets CD38, commonly expressed on abnormal clonal plasma cells^20^. Daratumumab may be administered intravenously (IV) or subcutaneously (SC), with the IV formulation historically associated with higher rates of infusion-related reactions (IRRs)^21^. In daratumumab clinical trials, IRRs were reported in approximately 37% of patients during the first IV infusion, with lower rates observed with subsequent administrations^22^. The SC formulation, though assessed in a different patient population, has been associated with a lower overall incidence of IRRs, approximately 7% in pooled safety analysis^23^. Given their frequency and clinical relevance, IRRs represent a suitable post-approval safety endpoint for evaluating the performance of an EHR-integrated research platform in real-world practice. This pilot study sought to assess the quality of data on IRRs in patients receiving daratumumab when captured through an EHR-integrated research platform.

## Results

### Study enrollment and cohort representativeness

The study enrolled 82 patients between April 2024 and March 2025 at four independent, community oncology practices (Table 1). Nearly all patients completed the study, with three early discontinuations: one due to death, and two due to patient withdrawal.

**Table 1:** Baseline characteristics of enrolled participants

Characteristic	*N* = 82
Age, years (continuous), Median (IQR)	71.0 (63–75)
Age, years (categorical), *n* (%)	
<65 years	25 (30.5)
65 to <70 years	13 (15.9)
70 to <75 years	21 (25.6)
≥75 years	23 (28.0)
Sex, *n* (%)	
Female	34 (41.5)
Male	48 (58.5)
Race, *n* (%)	
White	58 (70.7)
Black or African American	13 (15.9)
Other or not reported/unknown^a^	11 (13.4)
Ethnicity, *n* (%)	
Hispanic or Latinx	12 (14.6)
Not Hispanic or Latinx	66 (80.5)
Not reported/unknown	4 (4.9)
Multiple myeloma status, *n* (%)	
Newly diagnosed	63 (76.8)
Relapsed/refractory	19 (23.2)
Baseline ISS staging, *n* (%)	
I	27 (37.0)
II	32 (43.8)
III	14 (19.2)
Missing	9
Baseline ECOG score, *n* (%)	
0	42 (53.2)
1	23 (29.1)
2	11 (13.9)
3+	3 (3.8)
Field marked as “not done”^b^	3

*SD* standard deviation, *IQR* interquartile range, *ISS* International Staging System, *ECOG* Eastern Cooperative Oncology Group.

^a^“Other” race category includes “Other: Hispanic”, “Other: Dominican”, “Other: Mexican American Indian”, and “Other: Palastenian [sic]”.

^b^Fields explicitly marked as “not done” were not considered missing.

Centralized patient identification services screened 353,602 unique patients who were scheduled for upcoming visits, using a combination of automated matching and human review. Two hundred seventy-eight patients were deemed eligible and another 1594 as “watching” (patients who did not meet any exclusion criteria but did not yet satisfy all inclusion criteria). Eighty-two patients chose to enroll and were treated on study, 57 of whom were surfaced to the sites by the centralized matching service. Black and Latinx patients comprised 26.5% of overall site populations, 37.6% of those patients eligible based on structured data alone (e.g., International Classification of Diseases [ICD] code), 39.9% of those eligible after human review, and 30.5% of those who ultimately enrolled (all proportions include patients with incomplete race/ethnicity data based on harmonized EHR data as of August 2025).

### Data completeness

A total of 28 fields (variables) are reported in this manuscript, including 16 fields for baseline characteristics and 12 fields for daratumumab administration characteristics. Among the 4568 data points across 82 patients relevant to the primary objective (participant baseline characteristics and daratumumab administration), 4541 (>99%) were collected, and 27 (0.59%; 95% CI: 0.40–0.87%) were missing.

Among the 82 participants, no missing data points were observed for 16 fields including demographics, vital signs, medical history, and disease characteristics. Similarly, there was no missingness for laboratory measurements. Missingness was only observed for investigator-assessed ISS stage (*n* = 9, 11.0%), and bone marrow plasma cell percentage (*n* = 4, 4.9%).

Missingness of daratumumab administration data are summarized in Table 2. Of the 12 fields used to describe daratumumab administrations, nine fields had no missing data points across all administrations. Among IV administrations (4 patients, 12 infusions), all four fields had no missing data points. Among SC administrations (78 patients, 228 doses), injection start time was missing for 1 dose, and anatomical location was missing for 10 doses. Among IV and SC administrations combined, 3 data points for IRR mitigation treatment(s) after daratumumab administration were missing.

**Table 2:** Missingness of IRR data elements among patients who received at least 1 administration of daratumumab

Characteristic	Daratumumab administration #1, n (%)	Daratumumab administration #2, n (%)	Daratumumab administration #3, n (%)
**Among patients who received at least 1 daratumumab IV administration**	***N*** **=** **4**	***N*** **=** **4**	***N*** **=** **4**
Infusion volume	0 (0.0)	0 (0.0)	0 (0.0)
Infusion start time	0 (0.0)	0 (0.0)	0 (0.0)
Infusion stop time	0 (0.0)	0 (0.0)	0 (0.0)
Infusion interruptions	0 (0.0)	0 (0.0)	0 (0.0)
Among patients who received at least 1 daratumumab SC administration	*N* *=* *78*	*N* = 76	*N* = 74
Injection start time	0 (0.0)	1 (1.3)	0 (0.0)
Anatomical location	5 (6.4)	3 (3.9)	2 (2.7)
**Among patients who received at least 1 daratumumab IV/SC administration**	***N*** **=** **82**	***N*** **=** **80**	***N*** **=** **78**
Received IRR mitigation treatment(s) before daratumumab administration	0 (0.0)	0 (0.0)	0 (0.0)
Received IRR mitigation treatment(s) after daratumumab administration	1 (1.2)	1 (1.3)	1 (1.3)
Date of daratumumab administration	0 (0.0)	0 (0.0)	0 (0.0)
Route of daratumumab (SC or IV)	0 (0.0)	0 (0.0)	0 (0.0)
Prescribed dose of daratumumab	0 (0.0)	0 (0.0)	0 (0.0)
Receipt of other agent in treatment regimen with daratumumab	0 (0.0)	0 (0.0)	0 (0.0)

*IRR* infusion-related reaction, *IV* intravenous, *SC* subcutaneous.

### EHR-to-EDC automated data transfer

Overall, there were 43,941 data points collected in the EDC. Ninety one percent of data points were eligible to be transferred via the EHR-to-EDC connector, with nine percent requiring manual site data entry into the EDC (e.g., End of Treatment form). A total of 31,611 data points were pushed across 3173 EHR-to-EDC session submissions (10.0 data points per submission). Table 3 shows the potential vs. actual use of EHR-to-EDC data transfer. Note that data points may be transferred more than once to reflect source data changes to the EDC, so that data points pushed may not equal total data points collected.

**Table 3:** Data transfer eligibility/volume by data category

Data category	Data points by eligibility category, n (column %)^a^	Data points by transfer status, n (column %)^b^
Structured	6428 (14.6)	5218 (11.9)
E-source forms	18,969 (43.2)	15,538 (35.4)
Unstructured	14,549 (33.1)	8669 (19.7)
Manual/direct site entry	3995 (9.1)	14,516 (33.0)
Total	43,941 (100)	43,941 (100)

*E-source forms* electronic source forms.

^a^Eligibility refers to data points that were mapped in the electronic health record (EHR)-to-electronic data capture (EDC) connector and were therefore potentially eligible for transfer via EHR-to-EDC connection.

^b^Transfer status refers to data points that were transferred using the EHR-to-EDC connector.

As timeliness of data acquisition is critical to study completion and analysis, we measured the timelines for data entry in the EDC, categorized by the type of data entry required. As shown in Table 4, the median time from scheduled visit to EDC data entry was lowest for those parameters that could be directly transferred by FHIR (1.7 days) and highest for those requiring centralized abstraction (9.1 days).

**Table 4:** Median (IQR) for days between scheduled visits and EDC data entry, by visit group^a^

Data entry mode	Overall	Enrollment	Treatment	End of trial/treatment	Unscheduled visits
Structured	1.7 (1.0–4.3)	1.6 (0.90–4.7)	1.6 (0.97–4.1)	–^b^	4.7 (2.8–4.7)
Unstructured	9.1 (4.9–19.8)	11.3 (6.7–20.8)	9.0 (4.2–19.1)	–	–
E-source forms	4.7 (1.7–13.2)	4.7 (1.7–13.2)	–	–	–
Manual/direct site entry	4.7 (1.2–16.5)	5.5 (0.90–15.8)	4.6 (1.3–20.5)	3.6 (0.85–10.5)	4.7 (2.8–4.7)
Overall	5.2 (1.7–14.8)	4.9 (1.2–14.3)	5.7 (2.0–15.1)		4.7 (2.8–4.7)

*EDC* electronic data capture, *E-source forms* electronic source forms, *IQR* interquartile range.

^a^Applies to visit case report forms and timed subject log data (i.e., Enrollment, Treatment Phase, End of Trial/Treatment, and Unscheduled Visits), but not unscheduled/ongoing subject logs.

^b^Empty cells indicate that this visit group had no data entered via this data entry mode.

### Data queries

In total, 5752 “field-level” queries (i.e., associated with a specific field in the EDC) were opened (Table 5). 5617 of the field-level queries were opened manually by the sponsor, with the remainder surfaced as automated edit checks. 4519 (80.5%) queries were resolved within two weeks with a median time to resolution of 5.1 days.

**Table 5:** Query metrics

Data category	Manual field-level queries, n (column %)	Manual field-level queries (excluding out-of-range), n (column %)	Data points collected, n^c^	Query-to-data point ratio (excluding out of range), %^c^	Queries resolved within 2 weeks, % (95% CI)^c^	Median time to resolution, d (IQR)^c^
Initially missing Data^a^	3973 (70.7)	3973 (76.3)	–^b^	–	79.9 (78.7–81.2)	5.1 (2.0–8.9)
Structured data	395 (7.0)	24 (0.46)	5218	0.5	85.6 (82.1–89.0)	4.8 (1.8–7.8)
E-source forms	318 (5.7)	318 (6.1)	15,538	2.0	83.3 (79.2–87.4)	5.1 (0.82–8.0)
Unstructured data	133 (2.4)	133 (2.6)	8669	1.5	71.4 (63.8–79.1)	6.9 (1.4–36.8)
Manual/direct site entry	798 (14.2)	762 (14.6)	14,516	5.2	81.0 (78.2–83.7)	4.1 (0.85–10.1)
Total	5617 (100)	5210 (100)	43,941	–	80.5 (79.4–81.5)	5.1 (1.9–8.9)

*E-source forms* electronic source forms.

^a^Data may have eventually been entered as the study progressed.

^b^Empty cells are not reported as they depend on data points collected and do not apply for missing data.

^c^These metrics pertain to the data category, i.e., metrics are aggregated at the row level.

## Discussion

This pilot study demonstrated that a technology-enabled research infrastructure, integrating real-time patient identification, structured EHR-to-EDC data transfer, and centralized abstraction services can be operationalized in community oncology settings to support prospective post-approval evidence generation. The study was designed with operational and regulatory elements required to fulfill a PMC, including alignment with FDA data standard guidance^24^ and guidance regarding the use of EHR data in clinical investigations^25^. Overall, data missingness was low (0.59%). Most structured data fields were successfully mapped from the EHR into the EDC system, while additional unstructured fields were populated through centralized abstraction. These processes reduced the reliance on site-based transcription and contributed to low rates of missingness, particularly for protocol-defined variables related to IRRs.

Eighty-two patients with multiple myeloma were accrued to this study at four community oncology sites over an 11-month period. This rapid accrual was associated with a centralized technology-enabled patient identification service, designed to reduce site burden. Furthermore, 30.5% of enrolled patients identified as Black or Hispanic, far higher than other recent studies of patients with myeloma^26^. This higher enrollment of Black and Hispanic patients is likely attributable in part to the intentional inclusion of study sites that serve diverse populations. Together these results raise the possibility that automation may improve patient ascertainment, mitigate unconscious bias associated with patient selection, and advance trial representativeness. However, a direct comparison of the race and ethnicity of patients who enrolled to those who did not enroll is hindered by increased missingness in the availability of self-reported demographic data among patients who did not participate in this study. In addition, we did not capture reasons for non-enrollment of patients who were surfaced as potentially eligible. For these reasons, any conclusions regarding enrollment representativeness should be viewed as exploratory and hypothesis-generating.

All 82 enrolled participants received daratumumab, and nearly all (79/82) completed study follow-up, supporting the feasibility of prospective safety data collection in a routine care environment. Importantly, the platform supported participation from community-based centers, which often have limited engagement in research due to administrative and infrastructure constraints. The ability to capture high-quality data in these settings illustrates the potential for broader inclusion in regulatory research studies, particularly those designed to contribute generalizable safety data post-approval.

Delays in data submission from study sites and query resolution impede the pace of evidence generation^27–29^. In this study, the time from patient visit to data receipt by the EDC was less than 1 week (median 5.2 days) overall, with a median time of 1.7 days for structured data transferred through the EHR-to-EDC connector. Furthermore, the proportion of manually opened field-level queries per datapoint was ≤2% among EHR-to-EDC transferred data (excluding out-of-range lab and vitals results). This rate is significantly lower than the 5.2% observed for elements populated manually by site staff, suggesting the potential to reduce transcription errors significantly. Query resolution was rapid, with a median of 5.1 days. Together, these metrics suggest a high-level efficiency for clinical staff associated with centralization and automation of research processes, and rapid receipt of data for analysis.

Additional operational efficiencies associated with this EHR-based platform included a reduction in the burden of data entry at clinical sites, streamlined monitoring workflows, and minimized query volume, driven in part by structured EHR-to-EDC data transfer and centralized source verification. While traditional prospective clinical studies rely on site-based transcription and monitoring processes, this approach allowed for scalable, centralized operations with lower disruption to clinic workflows. Given the association of site research participation with high-quality care, this EHR-based platform with centralized services implemented in community practice settings could have broader implications for patient care^30^.

In this pilot study, we observed incomplete use of the EHR-to-EDC connector with 67% of data elements transferred vs. 91% eligible for transfer. It is plausible that the learning curve associated with adoption of new technology in a clinical setting is in part responsible for this finding^18,31^. Additionally, we discovered specific technical and workflow factors that at least partially contributed to incomplete automated transfer. Fields meant for abstraction took more time to populate in the EDC and did not involve site intervention, creating ambiguity about whether sites should enter these data themselves. Furthermore, laboratory tests that were not performed were unable to be transferred as such by the EHR-to-EDC tool. Users were required to manually report that the laboratory test was not performed in the EDC. Qualitative feedback from the study operational team and study sites indicated that additional site training and improved timeliness of centralized abstraction contributed to increasing use of automated data transfer during the course of the study. However, it was not feasible to quantitatively describe this trend within the scope of this study because of differences in site activation timelines and data cleaning operations (which encouraged manual data entry due to the nature of EDC query workflows).

The results we present must be interpreted in the context of several potential limitations. Since this pilot study was conducted in only four clinical sites, the data may not be generalizable to all community oncology practices or in the non-oncology context. In addition, we could not identify a published industry standard for data completeness or query frequency; these results are therefore not comparative and may be considered as a baseline for future research.

These results provide initial support for the use of EHR-integrated platforms in generating regulatory grade evidence in oncology. This platform was associated with robust and representative enrollment while reducing site operational burden. Support for timely data analysis was provided by a low-level of missing data and data queries, with rapid receipt of data in the study database following patient encounters. Expanding clinical research from traditional academic trial sites to the community oncology setting can accelerate the generation of generalizable evidence. While this pilot study was observational, this platform is relevant to interventional studies, with use-cases that include regulatory PMRs, expanded indications for cancer therapeutics, as well as non-regulatory evidence needs that inform routine practice.

## Methods

### Study design

The overall goal of this pilot study was to evaluate the feasibility of an EHR-integrated platform for post-approval safety monitoring. This was a non-interventional, prospective pilot study conducted across four US community practice clinical sites. The study was designed to evaluate data quality performance of an EHR-integrated data collection infrastructure to capture incidence and characteristics of severe (Grade 3–4) and fatal (Grade 5) IRRs in patients with multiple myeloma receiving daratumumab. Data were captured before and after the first three administrations of daratumumab, administered per routine clinical care and in accordance with approved local practice. Data quality was assessed based upon data completeness and query frequency.

The study was performed in accordance with the Declaration of Helsinki. Written informed consent was obtained from all participants, authorizing the use of source data for research and allowing verification in compliance with applicable local regulatory and ethical requirements. Institutional review board approval of the study protocol was obtained from WCG IRB (reference number: IRB00000533).

### Objectives

This pilot study aimed to assess an EHR-based data collection infrastructure for capturing potential risk factors for severe and fatal IRRs in participants treated with IV or SC daratumumab for the treatment of multiple myeloma in the community practice setting. Protocol-specified data quality and objectives were:

1. To assess the completeness of study data collected from the EHR and transmitted to the EDC via an EHR-to-EDC connector (primary objective). Relevant endpoints were missingness of IRR risk factor variables in EDC for all enrolled participants and missingness of IRR data elements in EDC for participants who received at least 1 administration of daratumumab.
2. To describe operational time and effort of data collection and processing. Relevant endpoints were number of manual queries issued in the EDC, time to query resolution (personnel-related), time to EDC data availability after a study visit, data volume pushed per each EHR-to-EDC user session, percent of EDC data populated via EHR-to-EDC connector (data collection infrastructure-related).

In addition to these outcomes, in this manuscript we also present data on the efficiency of patient screening and associated cohort representativeness.

The results pertinent to clinical objectives regarding incidence and management of IRRs will be reported in subsequent publication.

### Eligibility criteria

Eligibility criteria were pragmatically designed^32,33^, consistent with routine use of daratumumab. Eligible subjects were at least 18 years old, with a diagnosis of multiple myeloma. Planned treatment with daratumumab in accordance with the approved label either as monotherapy or in combination with other agents was required. Patients with newly diagnosed multiple myeloma who were eligible for transplantation, and prescribed daratumumab according to National Comprehensive Cancer Network (NCCN) guidelines^34^ were also eligible. Patients who had previously received daratumumab or anti-CD38 antibody therapy or had received an investigational drug or medical device within two weeks of study start were excluded. Patients who were actively enrolled in an interventional clinical trial were not eligible.

### EHR-based operational innovations

**Fig. 1:**
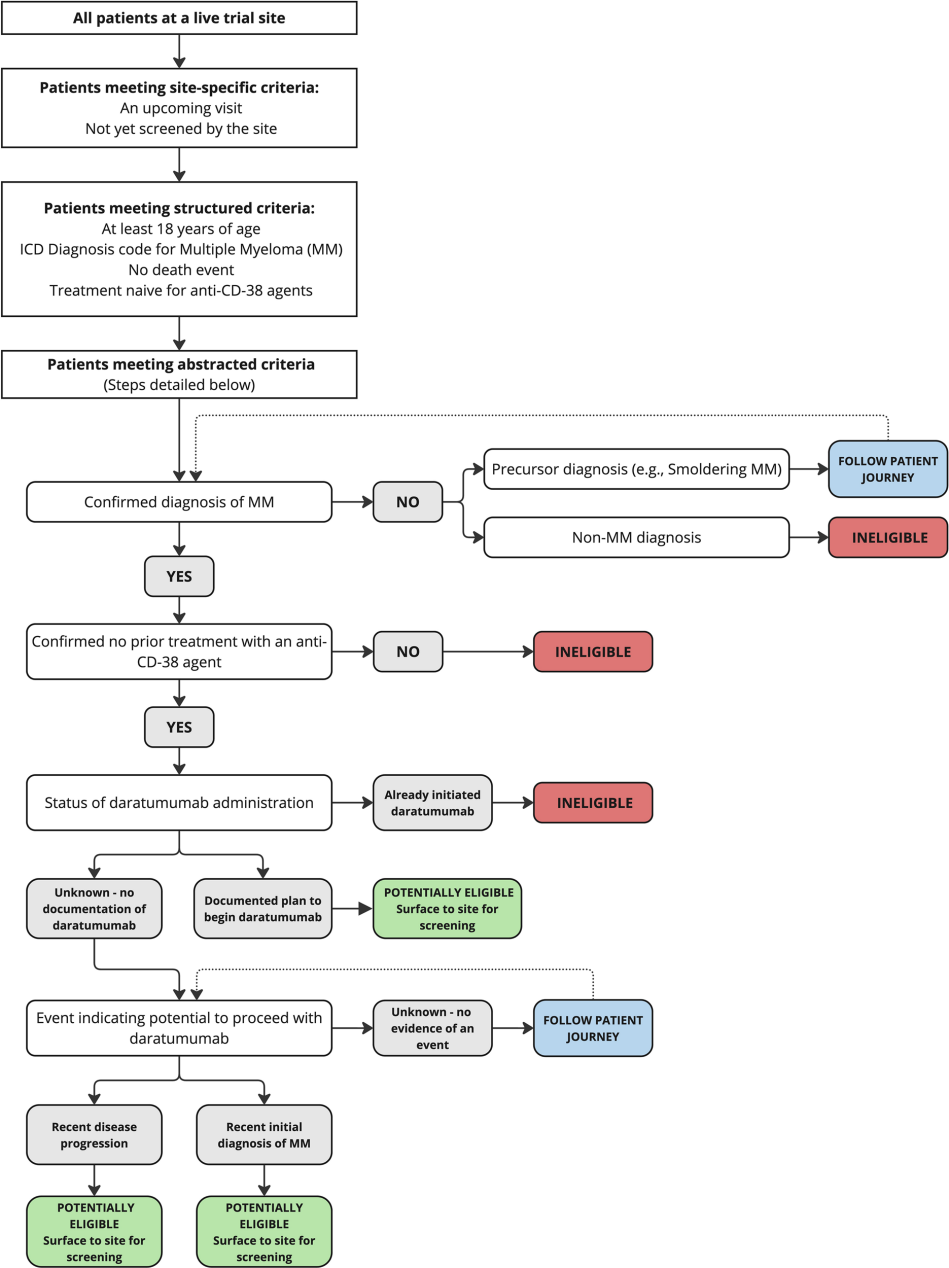
Patient selection process. MM multiple myeloma.

The study used a multi-component technology stack to support automated patient matching and data acquisition:

Patient identification and matching: A data driven approach was used to identify eligible participants, leveraging custom trial matching algorithms based on available structured data in the patient chart, and clinical review using a combination of specially-trained human abstractors and assistive technology. Site-specific and structured criteria were evaluated daily, and patients were considered potentially eligible if they: had an upcoming visit at a study site; were at least 18 years of age; had an ICD-10 code for multiple myeloma; had no death event from EHR, obituary, or Social Security Death Index data; and had no evidence of a drug order for anti-CD-38 agents. Potentially eligible patients were rereviewed by human abstractors when new documents were added to the patient chart^35^. All potential trial patients were then surfaced directly to site investigators and research staff directly at the point of care via an EHR-embedded interface^35^. A visual representation of the data flow architecture is provided in Fig. 1.

EHR-to-EDC data transfer: A secure EHR-to-EDC connector (Flatiron Clinical Pipe) was used to enable direct data transfer from the EHR system to the EDC platform, to reduce the need for manual data transcription. Site Initiation Visits included mandatory EHR-to-EDC connector training prior to data entry and subject enrollment. Sites received ongoing support throughout the study, including a comprehensive Data Entry Guide and access to a dedicated help desk to encourage effective implementation. Study-specific mapping was performed in the EHR-to-EDC connector to categorize study data into the following data transfer eligibility categories:

1. Structured data: includes elements such as lab observations and vital signs, transferred using Health Level 7 (HL7) Fast Healthcare Interoperability Resources (FHIR) standards.
2. Unstructured data: includes elements contained in clinician narratives and clinical notes that are relevant to the study’s objectives and endpoints. Examples include documentation of signs and symptoms, IRRs, pre- and post-administration medications, and provider-reported impressions. Source documents from the EHR are ingested via FHIR application programming interface into the EHR-to-EDC connector, which are then surfaced in an abstraction tool. This is a software tool that efficiently organizes and displays unstructured documents to trained human abstractors to capture key data elements in data entry forms. Centralized abstraction by trained personnel minimized site level variability in unstructured data interpretation. Edit Checks or automated discrepancy checks (e.g., start date later than end date) and routine monitoring enhanced consistency and accuracy.
3. Electronic (E)-Source Forms: these forms accessed through the EHR user interface and linked to EHR source data permit clinical trial-specific clinical information for study participants to be entered within routine workflows, largely as structured fields. Parameters included AE grading per Common Terminology Criteria for Adverse Events (CTCAE) version 5.0^36^, relevant past medical/surgical history, and concomitant medications.

The data transfer workflow involved launching the EHR-to-EDC connector from within the EHR user interface. The user (site staff) was then able to select which data to submit and transfer directly into the corresponding electronic case report form (CRF) within the EDC, via “point and click.” Live site staff training was conducted before study initiation. Sites were responsible for ensuring all relevant source data were populated in the EDC for monitoring or audit purposes. Data points could only be transferred according to their mapped data eligibility category and associated transfer mechanism; however, site staff could directly enter any data point into the EDC if preferred.

### Data standards and mapping

Study data mapping began with the creation of a data model & form- and field-level data dictionary. To ensure that the data collected would be suitable for a regulatory use case, the data elements were mapped for equivalence to an ongoing post-approval safety study^37^. After collection, all study data were mapped to the standard Clinical Data Standards Interchange Consortium (CDISC) Study Data Tabulation Model (SDTM), to ensure standardization, harmonization and suitability for regulatory submission of study data (to establish confidence in the application of this platform for future regulatory submissions).

### Quality control and monitoring

Data quality processes followed an integrated data review plan which included a cross-functional review across the clinical data management team, site monitors, medical monitors and clinical operations. A comprehensive quality control and monitoring framework was centered around evaluation of:

- Data missingness. Missingness is defined as any absence of a required variable in the EDC. Missingness could result from (1) lack of documentation at the source or (2) failure of data transfer into the EDC. Fields explicitly marked as “not done” are not considered missing.
- Query volume and median time to query resolution. As per Good Clinical Data Management Practices (GCDMP), a query is defined as a communication tool used to clarify and resolve discrepancies, inconsistencies, or missing information identified within the collected data of a clinical trial via manual and automatic system checks^38^. We measured the frequency of data queries and the timeliness of their resolution to elucidate efficiencies enabled by automated EHR-to-EDC data transfer. Time to query resolution is defined as the time (days) from when the sponsor initiated a query communication to the time when the sponsor marked the query as resolved. Multiple query communications on the same data point are possible.

Centralized abstraction workflows were led by data entry guidelines which define the expectations for data entry into the EDC system or completion of the CRF for the Principal Investigators, Site Coordinators, and designees. Select metrics were aligned with risk-based monitoring and Risk Assessment Categorization Tools (RACT)^39,40^.

This study’s EDC system used a commonly employed rules-based approach (or “Edit Checks”) for identification of discrepant data and had functionality for authoring, storing, managing, executing the rules and tracking the lifecycle of identified discrepancies.

### Variable definitions and data sources

All exposures, outcomes, and risk factor variables related to study endpoints were predefined and standardized using the study data dictionary. Data sources included both structured fields and unstructured clinical notes in the EHR, verified through abstraction and mapping workflows.

For variables with more than one data source (e.g., medications available via structured fields and physician notes), harmonization and cross-source validation were applied. Diagnostic criteria for IRRs were based on CTCAE v5.0^36^. Variables were categorized and transformed where needed (e.g., age categories, medication windows) based on clinically relevant groupings.

### Statistical analyses

The study was initially designed with a sample size of 50 patients, as a convenience cohort. To increase precision of outcome estimates and supported by rapid enrollment, the decision was made to extend the enrollment period an additional three months (final *n* = 82). All analyses were descriptive, and no formal hypothesis testing was planned. Categorical variables were summarized using frequencies and percentages, while continuous variables were described using median and interquartile range.

Baseline characteristics and the number and percentage of missing characteristics were summarized among enrolled patients. Missingness was defined as patients who do not have the characteristics or values of the characteristics available in the EDC. Missingness may occur due to data not being available at the source or data not being transferred from the source into the EDC. Characteristics include baseline demographics, vital signs, medical history, disease characteristics, and laboratory measurements. The number and percentage of missing IRR data elements are summarized per daratumumab administration among patients who received at least one administration. Characteristics of daratumumab administration include route of administration, prescribed dose, administration date, start time, pre- and post-medications, infusion volume/interruptions/stop time (for IV administrations), and anatomical location of administration (for SC administrations).

## Data Availability

Data sharing is not applicable to this article as no datasets were generated or analyzed during the current study.
